# Community-based screening for obstetric fistula in Nigeria: a novel approach

**DOI:** 10.1186/1471-2393-14-44

**Published:** 2014-01-24

**Authors:** Özge Tunçalp, Adamu Isah, Evelyn Landry, Cynthia K Stanton

**Affiliations:** 1 Department of Population, Family and Reproductive Health, Johns Hopkins Bloomberg School of Public Health, 615 N Wolfe St, Baltimore, MD 21205, USA; 2 EngenderHealth, Sokoto, Sokoto State, Nigeria; 3 EngenderHealth, 440 Ninth Avenue, New York, NY 10001, USA

**Keywords:** Fistula, Genito-urinary, Recto-vaginal, Prolapse, Community, Backlog, Africa, Program

## Abstract

**Background:**

Obstetric fistula continues to have devastating effects on the physical, social, and economic lives of thousands of women in many low-resource settings. Governments require credible estimates of the backlog of existing cases requiring care to effectively plan for the treatment of fistula cases. Our study aims to quantify the backlog of obstetric fistula cases within two states via community-based screenings and to assess the questions in the Demographic Health Survey (DHS) fistula module.

**Methods:**

The screening sites, all lower level health facilities, were selected based on their geographic coverage, prior relationships with the communities and availability of fistula surgery facilities in the state. This cross-sectional study included women who presented for fistula screenings at study facilities based on their perceived fistula-like symptoms. Research assistants administered the pre-screening questionnaire. Nurse-midwives then conducted a medical exam. Univariate and bivariate analyses are presented.

**Results:**

A total of 268 women attended the screenings. Based on the pre-screening interview, the backlog of fistula cases reported was 75 (28% of women screened). The backlog identified after the medical exam was 26 fistula cases (29.5% of women screened) in Kebbi State sites and 12 cases in Cross River State sites (6.7%). Verification assessment showed that the DHS questionnaire had 92% sensitivity, 83% specificity with 47% positive predictive value and 98% negative predictive value for identifying women afflicted by fistula among women who came for the screenings.

**Conclusions:**

This methodology, involving effective, locally appropriate messaging and community outreach followed up with medical examination by nurse-midwives at lower level facilities, is challenging, but represents a promising approach to identify the backlog of women needing surgery and to link them with surgical facilities.

## Background

Obstetric fistula is a complication that arises from prolonged or obstructed labour without prompt medical care which causes tissue necrosis resulting in a hole between the vagina and bladder or rectum, or both [1]. This compression and loss of blood supply produces necrosis of the compressed tissues resulting in uncontrolled leakage of urine from the bladder through the vagina, in the case of vesico-vaginal fistula (VVF) and leakage of stool from the vagina, in the case of recto-vaginal fistula (RVF) [1]. Frequently it results in a stillbirth and the woman is left with chronic incontinence leading to social problems such as rejection, shame, and stigma as well as economic problems [2]. Fistula can also result from sexual violence, complications from pelvic surgery or from harmful traditional practices such as *yankan gishiri,* a form of genital cutting practiced among the Hausa communities in Nigeria [3,4]. The underlying factors contributing to obstetric fistula include a dearth of skilled birth attendants, poverty, poor health seeking behaviour, poor referral systems and transportation network, and inadequate facilities providing comprehensive obstetric care services [5]. It is a condition that has been essentially eradicated in high-income countries where access to and quality of obstetric care are readily available [1]. However in many low-resource settings, it continues to affect women across a range of age groups and parities and have devastating effects on the physical, social, and economic lives of thousands of women [3,6,7].

An integrated approach to address fistula rests on three pillars of action: prevention, treatment and reintegration to the community [3,5]. Currently, Fistula Care, managed by EngenderHealth and supported by USAID, supports fistula treatment and prevention activities in 10 countries, including Nigeria [8]. In Nigeria, according to the 2008 Demographic and Health Survey (DHS), estimated prevalence of fistula symptoms in the southern zones ranges between 0.2% and 0.5% of reproductive aged women, and in the northern zones ranges between 0.3% and 0.8% [9]. The true prevalence and incidence of obstetric fistula remain difficult to determine for several reasons: lack of large-scale, prospective, population-based studies examining pregnancy outcomes (in order to measure incidence); few large, retrospective population-based studies of fistula prevalence; and, where smaller scale studies of fistula incidence and prevalence have been conducted, inaccurate measurement, due to problems regarding questionnaire design (inappropriate contingency questions or lack of specificity in the definition of fistula), or underreporting of fistula symptoms by women (due to the stigma associated with the condition) [10-12].

To effectively plan for the treatment of fistula cases, the Ministry of Health and its international partners need credible estimates of the backlog of existing cases requiring care. Backlog refers to the number of women who are currently living with fistula and can be expected to come to facilities for repair services. In 2008, the South East Regional Vesico Vaginal Fistula Centre (currently renamed the National Obstetric Fistula Centre, Abakaliki) conducted community screenings in 13 Local Government Areas (LGA) in Ebonyi State [13]. Over two months in 2008, a total of 559 women were screened of which 306 (54.7%) were diagnosed with fistula and referred to facilities [13]. Building upon that experience, this study was incorporated into the on-going activities in two states (Kebbi and Cross River States).

The objectives of this study were to quantify the backlog of obstetric fistula cases within select LGAs of Kebbi and Cross River States via community-based screenings in these LGAs. We also assessed the questions in the Demographic Health Survey (DHS) fistula module by comparing women’s self-reported fistula symptoms to results from the medical assessment during the screenings.

## Methods

This cross-sectional study was conducted in three stages: Selection and training of the screeners, research assistants, providers and sites, pre-screening community outreach, and the screening procedures. The study protocol was reviewed and approved by Western IRB in the USA and National Health Research Ethics Committee in Nigeria.

### Study setting: selection of the screening sites and providers

**Figure 1:**
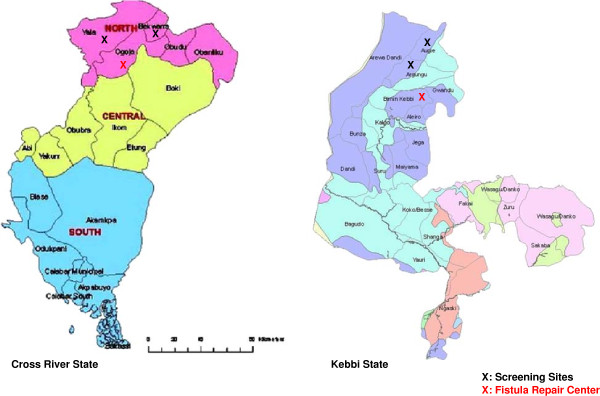
Maps of Cross River and Kebbi States and Screening Sites.

The screening sites were selected based on geographic coverage within the state (north and south), close relationships with the communities and availability of the fistula surgery facilities. The first state, Kebbi, is situated in the Northwest region with a majority Muslim population. EngenderHealth has been active in this state for more than ten years, including supporting a fistula centre since 2007. Two LGAs further identified were Argungu and Augie. Cross River State is situated in the South-south region with a majority Christian population. Fistula Care’s work is relatively new in this state, supporting a fistula centre since 2011. Two LGAs further identified were Bekwarra and Yala. The facilities, where the screenings took place were either a primary health care facility (PHC) or a general hospital (secondary health care facility), and they were assessed for their readiness; Fistula Care provided the materials needed during the examinations. Figure 1 shows the screening sites and the fistula centres where the women identified with fistula were referred.

Three experienced female nurse-midwives were trained on diagnosis and documentation of fistula before the screening. The nurses were trained for 10 days during a period, when surgeons from different hospitals convened for 5 to 10 days to perform more complex fistula surgeries [14]. The choice of nurse-midwives as screeners was an important part of our methodology, as we wanted to affirm that screening did not require fistula surgeons and/or obstetricians-gynaecologists. In addition we wanted to assess this approach to address cultural sensitivities in the predominately Muslim communities in Kebbi State where female providers may be more acceptable.

### Pre-screening community outreach

Fistula Care works in close relationship with local community-based organizations (CBOs) and has strong connections to the communities. Four weeks before the initiation of the screenings, outreach efforts were coordinated, which included advocacy and collaboration with the traditional leaders, village heads, LGA government staff, health educators and religious leaders. Table 1 summarizes the messages used in both of the states. It should be noted that as an outcome of outreach efforts, the officials in Kebbi LGAs provided transportation for the screenings, whereas this was not the case for screenings in Cross River LGAs.

**Table 1:** Community messages used pre-screening


Message 1	The continuous leakage of urine or faeces or both through woman’s private part is called a fistula.
Message 2	This condition can be completely treated through surgical operation in the hospital.
Message 3	The women who are identified with this condition at the screening will be operated free of charge.
Message 4	Women with other forms of leakage not related to fistula will be referred to other hospitals to get treatment, where they shall bear the cost of transportation and operations.

### Study population and screening procedures

The study included women who presented for fistula screenings at study facilities based on their perceived fistula-like symptoms. Although it is rare, women may become pregnant with a fistula, therefore we did not exclude pregnant women. There were no exclusion criteria.

The pre-screening questionnaire covered demographic information, including but not limited to date of birth, age, religion, marital status, highest educational attainment, community in which they live, number of live births, abortions and stillbirths. This questionnaire also included the set of questions from the fistula module used in the 2008 Nigeria DHS. Using data from these questions, we assessed self-reported fistula-like symptoms against data from the subsequent medical exam which involved use of a speculum and a dye test [1,9]. Questions regarding the community-based messages that were included in outreach efforts were asked, as well as questions pertaining to possible barriers to attending the screening. Following the Kebbi screenings we slightly modified the questionnaire to better discern how the specific community outreach messages were comprehended by the women in the communities and to better assess the degree of uterine prolapse, given the high number of prolapse cases identified in the Kebbi screenings. This questionnaire can be found as Additional file 1.

While the women were waiting to be screened our research assistants, trained on the study procedures in a 3-day workshop, invited the women to a private room to go over the written consent form to participate in the study, and among those who consented, administered the pre-screening questionnaire. All of the women who showed up at the screening were first interviewed using the questionnaire and received a physical examination by the nurse-midwives to assess their physical condition and to make an initial diagnosis on whether they had an obstetric fistula or some other condition. Results of the clinical exam were recorded by the nurse-midwife on the clinical examination form.

The women diagnosed with fistula were scheduled for surgery within 2–6 months, depending on caseload and surgeon availability. Of note, the definitive diagnosis of fistula was obtained during the examination while the woman was under anaesthesia. Women with conditions other than fistula were referred to local hospitals with the capacity to treat these conditions. The same team of research assistants and nurse-midwives participated in all of the screenings to ensure continuity and comparability.

### Data collection and analysis

Instead of hardcopy data collection, we utilized the free program called EpiData to directly enter women’s responses to the screening questionnaire on a computer [15]. This program allows implementation of alerts, error messages and skip patterns, and reduces the likelihood of entry errors as well as missing information. At the end of each day, data from the medical form (including the diagnosis) were also entered into EpiData by the research assistants. To ensure data quality, the data files were double-checked. The main outcome variables were the perceived fistula-like symptoms, the medical diagnosis of fistula in the pre-screening questionnaire and results from the medical exam. Univariate and bivariate analyses were conducted using Stata Version 12 [16].

## Results

A five-day screening was conducted in each selected LGA facility for both Kebbi and Cross River states for a total of 20 days. Overall, a total of 268 women attended these screenings: 88 in Kebbi State sites (July 9–20, 2012) and 180 in Cross River sites (November 27-December 7, 2012). All of the women gave consent to be part of the study.

In terms of socio-demographic characteristics, study participants from Cross River state were older, more likely to be widowed and with higher education relative to study participants in Kebbi (Table 2). In both of the states, almost all of the participants lived in the LGA where the screenings were conducted.

**Table 2:** Background characteristics of the study participants attending screenings in Kebbi state and Cross River State (N = 268)

	**Kebbi State (N = 88) n(%)**	**Cross River State (N = 180) n(%)**	**TOTAL (N = 268) n(%)**
*Age*			
Median (25%, 75%)	30 (23,40)	39.5 (28, 55)	35 (26, 50)
*Religion*			
Christian	0 (0)	179 (99.4)	179 (66.8)
Muslim	88 (100)	1 (0.6)	89 (33.2)
*Marital status*			
Married/Cohabitating	54 (61.4)	98 (54.4)	152 (56.7)
Divorced/Separated	19 (21.6)	25 (13.9)	44 (16.4)
Widowed	13 (14.7)	47 (26.1)	60 (22.4)
Single	2 (2.3)	10 (5.6)	12 (4.5)
*Education*			
None	80 (90.9)	101 (56.1)	181 (67.5)
Primary	5 (5.7)	47 (26.1)	52 (19.4)
Secondary	3 (3.4)	21 (11.7)	24 (9.0)
More than secondary	0 (0)	11 (6.1)	11 (4.1)
*LGA*			
Argungu	39 (44.3)	n/a	39 (14.5)
Augie	48 (54.5)	n/a	48 (17.9)
Other-Kebbi	1 (1.2)	n/a	1 (0.37)
Bekwarra	n/a	82 (45.6)	82 (30.6)
Yala	n/a	92 (51.1)	92 (34.3)
Other-Cross River	n/a	6 (3.3)	6 (2.2)
*Ever had fistula-like symptoms*			
No	38 (43.2)	151 (83.9)	189 (70.5)
Yes	50 (56.8)	29 (16.1)	79 (29.5)

In terms of fistula-like symptoms, 50 women (56.8%) in Kebbi State screenings reported ever having fistula-like symptoms compared with 29 women (16.1%) women in Cross River state screenings. The fistula-like symptoms were explored further among these women using data from the questions from the DHS fistula module. Table 3 summarizes the results. In both states, one third of women report having the symptoms between 1–5 years, with the majority reporting them after delivery, specifically after a difficult labour. Eleven per cent of the women in Kebbi report arriving at the facility after less than 12 hours of labour, whereas in Cross River 26% report arriving at the facility in less than 12 hours. The participants in Cross River State report a higher percentage of C-section for the delivery that resulted in their reported fistula-like symptoms than those in Kebbi, 80% and 56% respectively. The majority of women report having a stillbirth as an outcome, 84% and 74% in Kebbi and Cross River States respectively. The majority of the women in both of the states sought treatment from health care providers. Success rates after the latest treatment (not specified for surgery) was low, only one woman in the Kebbi State screenings and three women in the Cross River State screenings reported no more leakage at all.

**Table 3:** Fistula-like symptoms and related characteristics reported by the study participants attending Screenings in Kebbi State and Cross River State (N = 79)

	**Kebbi State (N = 50) n(%)**	**Cross River State (N = 29) n(%)**	TOTAL* (N = 79) n(%)
*Duration of the symptoms*			
Within the last 12 months	12 (24.0)	3 (10.3)	15 (19.0)
1–5 years	15 (30.0)	10 (34.5)	25 (31.7)
More than 5 years	23 (46.0)	16 (55.2)	39 (49.3)
*Precipitating event*			
After delivery	37 (80.4)	19 (73.1)	56 (77.8)
After some kind of illness	5 (10.9)	4 (15.4)	9 (12.5)
Spontaneous/Congenital	2 (4.3)	1 (3.8)	3 (4.2)
During pregnancy	2 (4.3)	0 (0.0)	2 (2.8)
After an operation	0 (0.0)	1 (3.8)	1 (1.4)
Don’t know	0 (0.0)	1(3.8)	1(1.4)
*Delivery*			
Normal labor/delivery	4 (10.8)	2 (10.5)	6 (10.7)
Difficult labor/delivery	33 (89.2)	17 (89.5)	50 (89.3)
*Delivery Location*			
Home	10 (27.0)	4 (21.1)	14 (25.0)
Hospital	27 (73.0)	15 (78.9)	42 (75.0)
*Arrival time at the facility after onset of labor*			
<12 hours	3 (11.1)	4 (26.7)	7 (16.7)
12–24 hours	13 (48.2)	5 (33.3)	18 (42.9)
>24 hours	10 (37.0)	6 (40.0)	16 (38.1)
Don’t know	1 (3.7)	0 (0)	1 (2.3)
*C-Section*			
No	12 (44.4)	3 (20.0)	15 (35.7)
Yes	15 (55.6)	12 (80.0)	27 (64.3)
*Stillbirth*			
No	6 (16.2)	5 (26.3)	11 (19.6)
Yes	31 (83.8)	14 (73.7)	45 (80.4)
*Treatment seeking for fistula-like symptoms*			
No	10 (20.0)	9 (31.0)	19 (24.1)
Yes	40 (80.0)	20 (69.0)	60 (75.9)
*Last treatment sought from*			
Health care provider	30 (75)	18 (90.0)	48 (80.0)
Untrained providers	10 (25)	2 (10.0)	12 (20.0)
*Success after the latest treatment*			
Yes, no more leakage at all	1 (2.5)	3 (15.0)	4 (6.7)
Yes, but still some leakage**	19 (47.5)	4 (20.0)	23 (38.3)
Still have problem***	20 (50.0)	13 (65.0)	34 (55.0)

*Denominators may change depending on the question as certain questions are only asked on depending on the previous responses.

**This refers to women who still report some kind of incontinence after the treatment they received.

***This refers to women who still have the fistula-like symptoms.

Table 4 presents results regarding women’s comprehension of the messages and outreach efforts. As can be observed, the penetration of the messages was mixed. Messages regarding the free-of-charge fistula surgeries were heard the least frequently (56%). The most frequently heard message (83%) related to the fact that women with other forms of leakage would be referred to other facilities at their own costs.

**Table 4:** Communication and access to the screenings in Kebbi State and Cross River State (N = 268)

	**Kebbi State (N = 88) n(%)**	**Cross River State (N = 180) n(%)**	**TOTAL (N = 268) n(%)**
Messaging*			
The continuous leakage of urine or feces or both through woman’s private part is called a fistula.	n/a	56 (65.9)	56 (65.9)
This condition can be completely treated through surgical operation in the hospital.	n/a	63 (74.1)	63 (74.1)
The women who are identified with this condition at the screening will be operated free of charge.	n/a	48 (56.5)	48 (56.5)
Women with other forms of leakage not related to fistula will be referred to other hospitals to get treatment, where they shall bear the cost of transportation and operations.	n/a	71 (83.5)	71 (83.5)
*Source of information about the screenings*			
Community organization	5 (5.7)	34 (18.9)	39 (14.5)
Radio spots	1 (1.1)	1 (0.6)	2 (0.7)
Family	19 (21.6)	17 (9.4)	36 (13.4)
Acquaintance/friend	8 (9.1)	7 (3.9)	15 (5.6)
Town crier	3 (3.4)	57 (31.7)	60 (22.4)
Village head	40 (45.4)	1 (0.6)	41 (15.3)
Church related staff	0 (0)	40 (22.2)	40 (14.9)
Health care staff	9 (10.2)	18 (10.0)	27 (10.1)
Other	3 (3.4)	5 (2.8)	8 (3.0)
Transportation to the screenings**			
On foot	5 (5.7)	55 (30.5)	60 (22.4)
Motorcycle	34 (38.6)	82 (45.5)	116 (43.3)
Car	52 (59.1)	38 (21.1)	90 (33.6)
Taxi/Public Moto	9 (10.2)	8 (4.4)	17 (6.3)
Bus	0 (0.0)	1.0 (0.5)	1 (0.4)
Other	0 (0.0)	1 (0.6)	1 (0.4)
*Accompaniment to the screenings*			
Alone	46 (52.3)	153 (85.0)	199 (74.2)
Husband	10 (11.4)	4 (2.2)	14 (5.2)
Mother/Father	7 (7.9)	4 (2.2)	11 (4.1)
Sister/Brother	4 (4.5)	7 (3.9)	11 (4.1)
Friend/Acquaintance	11 (12.5)	3 (1.7)	14 (5.2)
Other family	10 (11.4)	9 (5.0)	19 (7.1)

*This set of specific questions on the correct understanding of specific messages used in the communities during the outreach activities were added after the Kebbi screenings; therefore we only have data from the Cross River State screenings.

**Women might have reported more than one transportation method.

The channels through which women heard about the screenings differed between the two states. In Kebbi LGA screenings, almost half of the women heard about the screenings from the village heads, which was negligible in Cross River. Community organizations, town criers and churches were the main channels in the Cross River screenings. Transportation was another issue where the two states differed. In Kebbi LGAs almost 60% of the women came by car, whereas in Cross River LGAs 31% of the women were on foot and the rest used different types of vehicles, including motorcycle and car. In terms of accompaniment to the screenings, 85% of the women in Cross River LGAs were unaccompanied, whereas only 52% of the women in Kebbi LGAs came alone; among the accompanied women, most came with a family member (11% with a husband) or a friend.

**Figure 2:**
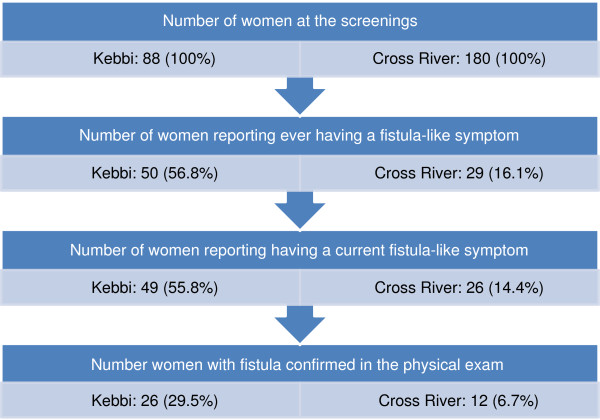
Flowchart of women at the screenings (N = 268).

Table 5 summarizes the results of the medical examination. Based on the medical exam, the backlog of fistula (urinary and rectal) cases across both states was 38 women (14% of women screened); 26 fistula cases were from Kebbi LGA sites and 12 cases in Cross River LGA sites (Figure 2). Cystocele/rectocele is the most common diagnosis in Kebbi State (39.8%) and is the second most common diagnosis in Cross River (5.6%), followed by uterine prolapse. However, in terms of absolute numbers, only 31 women in Cross River LGAs were diagnosed with some form of uro-gynaecological problem, whereas all 88 women in Kebbi LGAs had some form of uro-gynecological problem.

**Table 5:** Medical examination diagnoses in Kebbi State and Cross River State Screenings (N = 268)

	**Kebbi State (N = 88) n(%)**	**Cross River State (N = 180) n(%)**	**TOTAL (N = 268) n(%)**
Genitourinary fistula	23 (26.1)	10 (5.6)	33 (12.3)
Rectovaginal fistula	3 (3.4)	2 (1.1)	5 (1.9)
Stress Incontinence	6 (6.8)	2 (1.1)	8 (3.0)
Cystocele/Rectocele	35 (39.8)	10 (5.6)	45 (16.8)
Uterine prolapse	21 (23.9)	7 (3.9)	28 (10.5)
Other (non uro-gyn related)	0 (0.0)	149 (82.8)	149 (55.6)

We assessed self-reporting of fistula-like symptoms in the screening questionnaire against actual diagnosis of fistula. For this calculation the following three questions in the DHS module were used: ever had fistula-like symptoms, sought care and outcome of care. The number of women reporting current fistula-like symptoms was calculated by subtracting the women who reported receiving successful treatment (no leakage) from the women reporting ever having fistula-like symptoms. Thus, based on women’s self-reports to these questions, the backlog of fistula cases reported among the study population was 75 (28% of women screened). Among women reporting symptoms, our assessment shows that the questions had 92% sensitivity, 83% specificity with 47% positive predictive value and 98% negative predictive value (Table 6).

**Table 6:** Reporting of current fistula-like symptoms versus fistula diagnosis

	***Medical screening for fistula***		
***Current fistula-like symptoms reported***	**No**	**Yes**	**Total**
**No**	190	3	193
**Yes**	40	35	75
**Total**	230	38	268

## Discussion

According to our results, the backlog is 26 fistula cases in Kebbi State sites and 12 cases in Cross River State sites. Of note, as of July 1st, 2013, all women identified with fistula in both states have received fistula repair surgery. Moreover, these screening events identified related morbidities such as uterine prolapse, which cause significant stress for women [17].

An important limitation of this backlog calculation is the assumption that everyone with fistula within the two study LGAs presented at the screenings. Given this limitation, it was not possible to estimate minimal fistula prevalence for the states. Recently, two different methodologies have been explored to estimate prevalence of fistula in community settings using a key informant method and the sisterhood method [11,18]. Comparing lists of specific women identified by CBOs as having fistula-type symptoms against the list of women who present at fistula screenings (and are identified as a fistula case) remains a worthwhile exercise for estimating the backlog of fistula cases, where close collaboration with CBOs is possible.

Our results also show that this methodology proves to be a feasible approach for identifying a minimum estimate of the backlog of women needing surgery in the LGAs where the screenings were conducted. The methodology involves community outreach followed up with screening by nurse-midwives at lower level facilities. Women diagnosed with fistula are then linked to fistula centres in the state. This constitutes an extension to current programming where the majority of screenings take place in high level facilities by gynaecologists from within or outside the countries [3,19]. Data from the fistula repair centres in both states show that women who are identified in community screenings are more likely to have had fistula for a longer period of time (1–5 years versus less than one year), underscoring the importance of the community-based approach and its ability to reach a different population than that reached by the fistula repair centres.

During this study, we also verified the fistula questions used in the DHS. Our analysis showed that the DHS fistula questions when used for screening purposes among women with perceived fistula symptoms has 92% sensitivity, 83% specificity with 47% positive predictive value and 98% negative predictive value, suggesting that this questionnaire can be used as a pre-examination tool in such community screening programmes where only the women who are identified as potentially having fistula would be medically examined. It should be noted that these estimates of sensitivity, specificity and positive/negative predictive value do not represent the validity of DHS fistula module questions as the assessment was restricted to women with perceived fistula-like symptoms and not to a sample of women of reproductive age. Important to note is the need to specify the treatment received by the women in terms of surgery, which would have facilitated the differentiation between women having fistula-like symptoms or residual incontinence after a surgical repair versus any other non-surgical treatment attempts. This question was not included in the 2008 questionnaire used by Nigeria, however it has since been included in the revised obstetric fistula module [20].

This community-based screening approach is a good use of financial and human resources as the health care staff consist of nurse-midwives and the screenings are conducted at lower-level facilities rather than relying on senior providers at higher level facilities [21,22]. Programmatic recommendations resulting from this study are summarized in Table 7.

**Table 7:** Programmatic recommendations for community-based fistula screenings


Recommendation 1	Transportation should be an essential element of community-based fistula screening programs.
Recommendation 2	Stronger ties with communities and better messaging strategies are crucial for success in identifying backlog in community-based fistula programs.
Recommendation 3	Facilities providing fistula surgery and national fistula programmes (including training programs for surgeons) should consider a) the feasibility of incorporating prolapse repair surgery into the services they offer; b) the implications that such changes might make in how they operate on prolapse; and c) provision of appropriate treatment regimens for women with post-surgery leakages without directing attention away from established fistula services.

For this approach to be successful, it is essential to have community participation and ownership by community leaders and government officials. For example, in Kebbi State, government officials and community leaders provided transportation throughout the screenings and village heads were actively involved in disseminating the messages. In contrast, in Cross River, despite multiple requests and continuing communication, local government and community leaders did not provide transportation support for the screening activities. Coupled with the wide geographic distribution of hamlets in Cross River, the access for women with fistula-like symptoms was hindered. It should also be noted that EngenderHealth was active in Kebbi State before the Fistula Care project started whereas project activities in Cross River State started less than two years ago and focused on capacity development for medical services before engaging the communities regarding demand for fistula services.

Locally appropriate messaging is equally important to the success of this approach. Unlike in Hausa (spoken in Kebbi State), there is not one word for fistula in the languages spoken in the Cross River State. This led to community partners using “reproductive health problems” to describe the reason for the screenings rather than “fistula”. The vagueness of the message is apparent in our data as the majority of women in these LGAs presented at the screenings with other ailments. In Kebbi state all 88 women screened had some form of uro-gynaecological problems ranging from fistula to pelvic organ prolapse. Although the number of women presenting for screening was below expectation in both of the states, the screenings in Kebbi were more successful in terms of identifying the backlog of fistula patients needing surgery.

Our study also raises a question regarding how to manage other uro-gynaecological problems such as uterine prolapse as well as post-surgery residual incontinence. For example, all of the eight women diagnosed with “urinary incontinence” in the medical screening were post-fistula surgery patients. Also, pelvic organ prolapse appears to be a common problem among this population. Facilities providing fistula surgeries and national fistula programmes (including training programs for surgeons) should consider a) the feasibility of incorporating prolapse repair surgery into the services they offer; b) the implications that such changes might make in how they operate on prolapse; and c) provision of appropriate treatment regimens for women with post-surgery leakages without directing attention away from established fistula services. Integration of treatment for pelvic floor disorders into fistula services has resource and training implications. Moreover, we need to better understand the underlying mechanisms of incontinence following fistula surgery to effectively determine their cause and appropriate treatment [23].

## Conclusions

This community-based methodology, involving community outreach followed with physical exam by nurse-midwives at lower level facilities closer to where women live, is a promising approach to identify backlog of women needing surgery and linking them with surgical facilities. Lessons learnt from this and other studies could be applied to improving screening methodologies used for estimation as well as programming in low-resource settings.

## Competing interests

Dr. Özge Tunçalp and Dr. Cynthia Stanton conducted this work under Stanton-Hill Research LLC. Dr. Adamu Isah and Ms. Evelyn Landry are staff members at EngenderHealth.

## Authors’ contributions

OT and CS designed the study and the tools and AI and EL provided feedback. OT and AI conducted the study in Nigeria. OT conducted the analyses and wrote the first draft of the manuscript. AI, CS and EL contributed to interpretation of the results and editing of the manuscript. All authors read and approved the final manuscript.

## Pre-publication history

The pre-publication history for this paper can be accessed here:

http://www.biomedcentral.com/1471-2393/14/44/prepub

## Supplementary Material

Additional file 1 Pre-screening interview (Nigeria) data collection tool. Click here for file
