# Supplementary material for: Community-based screening for obstetric fistula in Nigeria: a novel approach
Source: BMC Pregnancy Childbirth. 2014 Jan 24;14:44. doi: 10.1186/1471-2393-14-44 (PMC3905156; doi:10.1186/1471-2393-14-44)
Supplement: Additional file 1 — Pre-screening interview (Nigeria) data collection tool. [file 1471-2393-14-44-S1.pdf]

August 13th, 2012
Version 6

PRE-SCREENING INTERVIEW (NIGERIA)

	**SCREENING ID NUMBER**	☐☐☐☐	
**#**	**QUESTIONS**		**SKIP**
	**BACKGROUND INFORMATION**		
**1.**	What is your age?	☐☐	
**2.**	Which religion do you belong to?	Christian……0<br>Muslim……1<br>Other……2<br>If Other, please specify________	
**3.**	What is your marital status?	Married/Cohabitating……0<br>Divorced/Separated……1<br>Widowed……2<br>Single……3	
**4.**	What is the highest degree of formal education that you attained? *(Not including Islamic/Arabic education)*	None……0<br>Primary……1<br>Secondary……2<br>More than Secondary……3<br>Don't Know/Missing……9	
**5.**	Which community do you currently live in?	________________	
**6.**	Which LGA do you currently live in?	Bekwarra……0<br>Yala……1<br>Abi……2<br>Akamkpa……3<br>Akpabuyo……4<br>Bakassi……5<br>Biase……6<br>Boki……7<br>Calabar Municipal……8<br>Calabar South……9<br>Etung……10<br>Ikom……11<br>Obanliku……12<br>Obubra……13<br>Obudu……14<br>Odukpani……15<br>Ogoja……16<br>Yakurr……17<br>IF OTHER, please specify the STATE……18<br>________________	
**7.**	How would you describe where you live?<br>*(RA responds based on the prior answers)*	Urban……0<br>Rural……1<br>Don't know……9	
	**REPRODUCTIVE HEALTH**		
**8.**	Now I would like to ask about all the births you have had during your life. Have you ever given birth before?	NO……0<br>YES……1	If NO, skip #12
**9.**	How many sons or daughters to whom you have given birth are alive?	SONS ☐<br>DAUGHTERS ☐	
**10.**	Have you ever given birth to a boy or a girl who was born alive but later died?	NO……0<br>YES……1	If NO, skip to #12
**11.**	How many boys or girls have died like this?	Boys dead ☐<br>Girls dead ☐	
**12.**	Have you ever lost a pregnancy? It might be spontaneously or when you or someone else had to do something to end the pregnancy.	NO……0<br>YES……1	If NO, skip to #14
**13.**	How many pregnancies have you lost in your lifetime?	☐	
**14.**	Some women have stillbirths, that is, they give birth in late pregnancy to a dead child.	NO……0<br>YES……1	If NO, skip to #16

	Have you ever had a stillbirth?		
**15.**	How many stillbirths have you had in your lifetime?		
	**FISTULA-LIKE SYMPTOMS (DHS)**<br>Sometimes a woman can have a problem such that she experiences a constant leakage of urine or stool from her vagina during the day and night. This problem usually occurs after a difficult childbirth, but may also occur after a sexual assault or after a pelvic surgery. This is called vesicovaginal fistula (VVF).		
**16.**	Have you ever experienced a constant leakage of urine or stool from your vagina during the day and night?	NO……0<br>YES……1	IF NO, skip to #31
**17.**	Did this problem occur within the last 12 months?	NO……0<br>YES……1	IF YES, skip to #19
**18.**	What year did this problem occur?		
**19.**	Did this problem occur after a delivery?	NO……0<br>YES……1	If YES, skip to #20
**b.**	Did this problem occur after an operation in your pelvic area? (pelvic surgery)	NO……0<br>YES……1	If YES, skip to #26
**c.**	Did this problem occur after some other event?	NO……0<br>YES……1<br>If OTHER, please specify________2	
	RA CONFIRMATION QUESTION FOR THE TOOL: Problem after delivery?	NO……0<br>YES……1	If NO, skip to #26
**20.**	Did this problem occur after a normal labor and delivery, or after a very difficult labor and delivery?	Normal Labor/Delivery……0<br>Very Difficult Delivery……1	
**21.**	Where did the delivery take place?	Home……0<br>Facility……1	If HOME, skip to #24
**22.**	How long after the labor pains began did you go to the facility?	<12 hours……0<br>12-24 hours……1<br>>24 hours……2<br>I don't know……9	
**23.**	Did you get a cesarean section at the facility?	NO……0<br>YES……1	
**24.**	Was this baby born alive?	NO……0<br>YES……1	
**25.**	After which delivery did this occur?	Delivery Number	
**26.**	How many days after (ANSWER TO QUESTION #19) did the leakage start?	Number of days after the precipitating event<br>(Enter 99, if more than 99 days)	
**27.**	Have you sought treatment for this condition?	NO……0<br>YES……1	If YES, skip to #29
**28.**	Why have you not sought treatment?<br>*(Multiple options available)*	Did not know how it could be fixed……0<br>Do not know where to go……1<br>Too expensive……2<br>Too far……3<br>Poor quality of care……4<br>Could not get permission……5<br>Embarrassment……6<br>If Other, please specify________<br>________7	
	RA CONFIRMATION QUESTION FOR THE TOOL: Treatment sought?	NO……0<br>YES……1	If NO, skip to #31
**29.**	From whom did you last seek treatment?	Health Professional<br>Doctor/Clinical Officer……0<br>Nurse/Midwife……1<br>Patient Attendant……2<br>Other Person<br>Untrained village doctor……3<br>Traditional Birth Attendants (TBA)……4<br>If Other, please specify________5	

**30.**	Did the treatment stop the problem?	YES, No more leakage at all……………………….0<br>YES, But still some leakage………………………..1<br>NO, Still have problem………………………………..2	
**31.**	Are there any (other) women in your household who suffer from vesicovaginal fistula/obstetric fistula?	NO……………………………………………………………0<br>YES…………………………………………………………..1	
**32.**	How many (other) women in your household suffer from vesicovaginal fistula/obstetric fistula?	Number [ ][ ]<br>Don't Know……………………………………………99	If NO, skip to #34
**33.**	Did she/they come or are they planning to come to the screening?	NO……………………………………………………………0<br>YES…………………………………………………………..1<br>Don't Know………………………………………………9	
	**COMMUNICATION**		
**34.**	How did you hear about the screening today?	The community organization……………………..0<br>The radio spots…………………………………………1<br>Family………………………………………………………2<br>Acquaintance/Friend…………………………………3<br>Town Crier………………………………………………..4<br>Fistula patients…………………………………………5<br>If Other, please specify_____________________6	
	**Please let me know if you remember hearing any of the following messages (between questions 35-38):**		
**35.**	The continuous leakage of urine or feces or both through woman's private part is called a fistula.	NO……………………………………………………………0<br>YES…………………………………………………………..1<br>Don't Know………………………………………………9	
**36.**	This condition can be completely treated through surgical operation in the hospital.	NO……………………………………………………………0<br>YES…………………………………………………………..1<br>Don't Know………………………………………………9	
**37.**	The women who are identified with this condition at the screening will be operated free of charge.	NO……………………………………………………………0<br>YES…………………………………………………………..1<br>Don't Know………………………………………………9	
**38.**	Women with other forms of leakage not related to fistula will be referred to other hospitals to get treatment, where they shall bear the cost of transportation and operations.	NO……………………………………………………………0<br>YES…………………………………………………………..1<br>Don't Know………………………………………………9	
**39.**	How did you get to the clinic today?<br>*(Multiple options available)*	On foot……………………………………………………0<br>By bicycle…………………………………………………1<br>By motorcycle……………………………………………2<br>By car………………………………………………………3<br>By taxi/public moto…………………………………..4<br>By train……………………………………………………5<br>By cart………………………………………………………6<br>By using animals such as donkey, camel etc..7<br>If Other, please specify_____________________8	
**40.**	Did you come to the screening by yourself?	NO……………………………………………………………0<br>YES…………………………………………………………..1	If YES, skip to the END
**41.**	Who accompanied you to the screening?	Husband……………………………………………………0<br>Mother/Father……………………………………………1<br>Sister/Brother…………………………………………….2<br>Friend/Acquaintance…………………………………3<br>If Other, please specify_____________________4	

THANK YOU VERY MUCH FOR YOUR TIME. NOW, I'LL TAKE YOU BACK TO THE WAITING AREA.

Name of the interviewer:
Date of the interview:

day	month	year
